# Scheme for the Differential Testing of Nerves and Muscles

**Published:** 1904-04

**Authors:** 


Scheme for the Differential Testing of Nerves and Muscles. For use in Diagnosis. By J. Montgomery Mosher, M. D., Clinical Professor of Insanity, Neurology and Electrotherapeutics, Albany Medical College. Octavo, 58 pages; illustrated. Albany: Brandon Printing Co. 1903. (Price, $1.00.)

The importance of electrodiagnosis in the prognosis of many nervous diseases is so well recognised and so universally accepted that any device for simplifying the practice is to be gladly welcomed. Very few physicians, however, take the necessary time or have the properly equipped apparatus for making such tests, and, if an electrodiagnosis is to be made, the services of a specialist are enlisted. Many of the simpler cases could be tested in the general practitioner's office and to those who so desire the volume of Mosher will be specially advantageous. An outline of the subject only is here given,—the details are to be sought for in the larger works of von Ziemssen, Lewandowski and others.

The author gives explicit directions for the application of electrodes in testing the various motor points of the muscles and nerves; also, a short description of the reaction of degeneration, and cleverly devised tables for recording the results of electro-examinations. The illustrations of the motor points are well executed and add much to the general usefulness of the book.

